# Comparison of proximal versus distal upper-limb robotic rehabilitation on motor performance after stroke: a cluster controlled trial

**DOI:** 10.1038/s41598-018-20330-3

**Published:** 2018-02-01

**Authors:** Yu-wei Hsieh, Keh-chung Lin, Ching-yi Wu, Tsai-yu Shih, Ming-wei Li, Chia-ling Chen

**Affiliations:** 1 Department of Occupational Therapy and Graduate Institute of Behavioral Sciences, College of Medicine, Chang Gung University, Taoyuan, Taiwan; 2 Healthy Aging Research Center, Chang Gung University, Taoyuan, Taiwan; 3 Department of Physical Medicine and Rehabilitation, Chang Gung Memorial Hospital, Linkou, Taiwan; 4 School of Occupational Therapy, College of Medicine, National Taiwan University, Taipei, Taiwan; 5 Division of Occupational Therapy, Department of Physical Medicine and Rehabilitation, National Taiwan University Hospital, Taipei, Taiwan; 6 Department of Physical Medicine and Rehabilitation, Sijhih Cathay General Hospital, New Taipei City, Taiwan; 7 Graduate Institute of Early Intervention, College of Medicine, Chang Gung University, Taoyuan, Taiwan

## Abstract

This study examined the treatment efficacy of proximal-emphasized robotic rehabilitation by using the InMotion ARM (P-IMT) versus distal-emphasized robotic rehabilitation by using the InMotion WRIST (D-IMT) in patients with stroke. A total of 40 patients with stroke completed the study. They received P-IMT, D-IMT, or control treatment (CT) for 20 training sessions. Primary outcomes were the Fugl-Meyer Assessment (FMA) and Medical Research Council (MRC) scale. Secondary outcomes were the Motor Activity Log (MAL) and wrist-worn accelerometers. The differences on the distal FMA, total MRC, distal MRC, and MAL quality of movement scores among the 3 groups were statistically significant (*P* = 0.02 to 0.05). Post hoc comparisons revealed that the D-IMT group significantly improved more than the P-IMT group on the total MRC and distal MRC. Furthermore, the distal FMA and distal MRC improved more in the D-IMT group than in the CT group. Our findings suggest that distal upper-limb robotic rehabilitation using the InMotion WRIST system had superior effects on distal muscle strength. Further research based on a larger sample is needed to confirm long-term treatment effects of proximal versus distal upper-limb robotic rehabilitation.

## Introduction

Most stroke survivors are burdened with significant physical dysfunction, and approximately 60% to 80% continue to have upper-limb (UL) motor deficits into the chronic phase of stroke that have a large effect on their daily life^1,2^. Developing effective rehabilitation interventions to maxmize UL motor recovery and functional independence of patients with stroke is therefore one of the top priorities in clinical practice and research^3,4^.

Robot-assisted therapy (RT) has emerged during the last decade as a novel rehabilitation approach to intensify UL motor function^5–8^. RT helps provide intensive, repetitive, and interactive training in a controlled environment to promote motor control and recovery of patients^9–14^. Although positive results of RT on motor outcomes have been noted^13–15^, there are disparate effects and heterogeneities between trials depending on the robotic types (eg, exoskeleton versus end-effector, or proximal versus distal approach), protocols, dosages, and problems of patients^15,16^.

Very few studies have directly compared the relative effects of different robotic devices. A recent systematic review^15^ investigated the effect of robotic types and reported a trend favoring end-effector rather than exoskeleton robotic devices on motor function. However, the superiority of treatment effect on the UL joints targeted by robotics remains unknown, especially for distal robotics^15^. Thus, comparative trials of different robotic types (eg, proximal versus distal robots) are warranted to tailor robot-aided UL rehabilitation to patient’s needs.

This study mainly compared the treatment effects of the InMotion ARM versus the InMotion WRIST robotic systems. The major difference between the 2 robotic devices is that the InMotion ARM focuses on training shoulder and elbow movements (ie, proximal UL), and the InMotion WRIST targets wrist and forearm movements (ie, distal UL). The proximal UL segments are critical for stability and transport of the arm, and the distal UL joints are mainly responsible for object manipulation and are important for performing daily activities^17,18^.

Motor control of the proximal UL and distal UL might be driven by different descending pathways^19^. The dorsolateral pathways (eg, corticospinal and rubrospinal tracts) are important for control of distal UL movements, and the ventromedial pathways (eg, reticulospinal, vestibulospinal, and tectospinal tracts) act more on the axial and proximal UL muscles and movements^20,21^. Although the neural bases act on proximal and distal UL segments and their functional roles appear to be different, direct comparisons of the clinical efficacy of proximal versus distal UL training in stroke patients are lacking.

Mazzeloni *et al*.^22^ used the same robotic systems to evaluate the treatment effects of proximal RT versus distal RT and proximal RT combined in 2 groups. However, the study goals of Mazzeloni *et al*. and this work are different. The effects of RT directly related to the UL segments specifically treated could not be drawn from the study findings of Mazzeloni *et al*. The 2 RT systems, InMotion ARM and InMotion WRIST, allow us to directly compare the outcomes affected by the proximal versus distal UL training.

In addition, recent reviews of RT have shown non-significant improvements or small effects on daily function after UL robotic rehabilitation in patients with stroke^14,15,23^. Major goals of stroke rehabilitation are to improve not only motor function but also functional performance on daily activities. Moreover, many patients were unable to translate the improvements of motor function and muscle strength to daily activity performance, which led to persistent functional dependence^24^. Therefore, this study provided functional task practice after RT to enhance the gains from proximal and distal UL robotic rehabilitation on motor function and muscle strength transfer into the patients’ daily functional performance.

The study purposes were to investigate the treatment effects of proximal-emphasized RT by using the InMotion ARM (P-IMT) versus distal-emphasized RT by using the InMotion WRIST (D-IMT) compared with a control treatment (CT) in patients with stroke. We designed a conventional rehabilitation program as the CT to provide a higher-level of clinical evidence, which decreased the influence of nondirective research environment and participant factors on treatment efficacy (eg, the Hawthorne effect), and to pose a more ethical approach instead of no treatment or placebo.

## Methods

### Participants

The inclusion criteria were (1) first-ever unilateral stroke; (2) at least 6 months after stroke onset; (3) a baseline score on the Fugl-Meyer Assessment (FMA) of 18 to 56; (4) able to follow the study instructions (≥24 points on the Mini-Mental State Examination)^25^; and (5) no excessive spasticity in UL joints (modified Ashworth scale ≤ 3). The exclusion criteria were (1) severe neuropsychologic problems (eg, global aphasia or neglect); (2) other neurologic diseases; and (3) obvious joint pain or UL fracture within 3 months.

The Institutional Review Boards of the Chang Gung Memorial Hospital (IRB#103-3564A3), Buddhist Taipei TzuChi General Hospital (IRB#03-M03-055), Cathay General Hospital (IRB#CGH-P101054), Cheng Hsin General Hospital (IRB#(363)102A-11), and Taipei Hospital, Ministry of Health and Welfare (IRB#TH-IRB-0014-0004) in Taiwan approved the study. Two participating sites used the same IRB approval (ie, IRB#103-3564A3). All participants provided written informed consent. This study was conducted in accordance with the Declaration of Helsinki and the International Conference on Harmonization Guidelines for Good Clinical Practice.

### Study design and procedure

**Figure 1:**
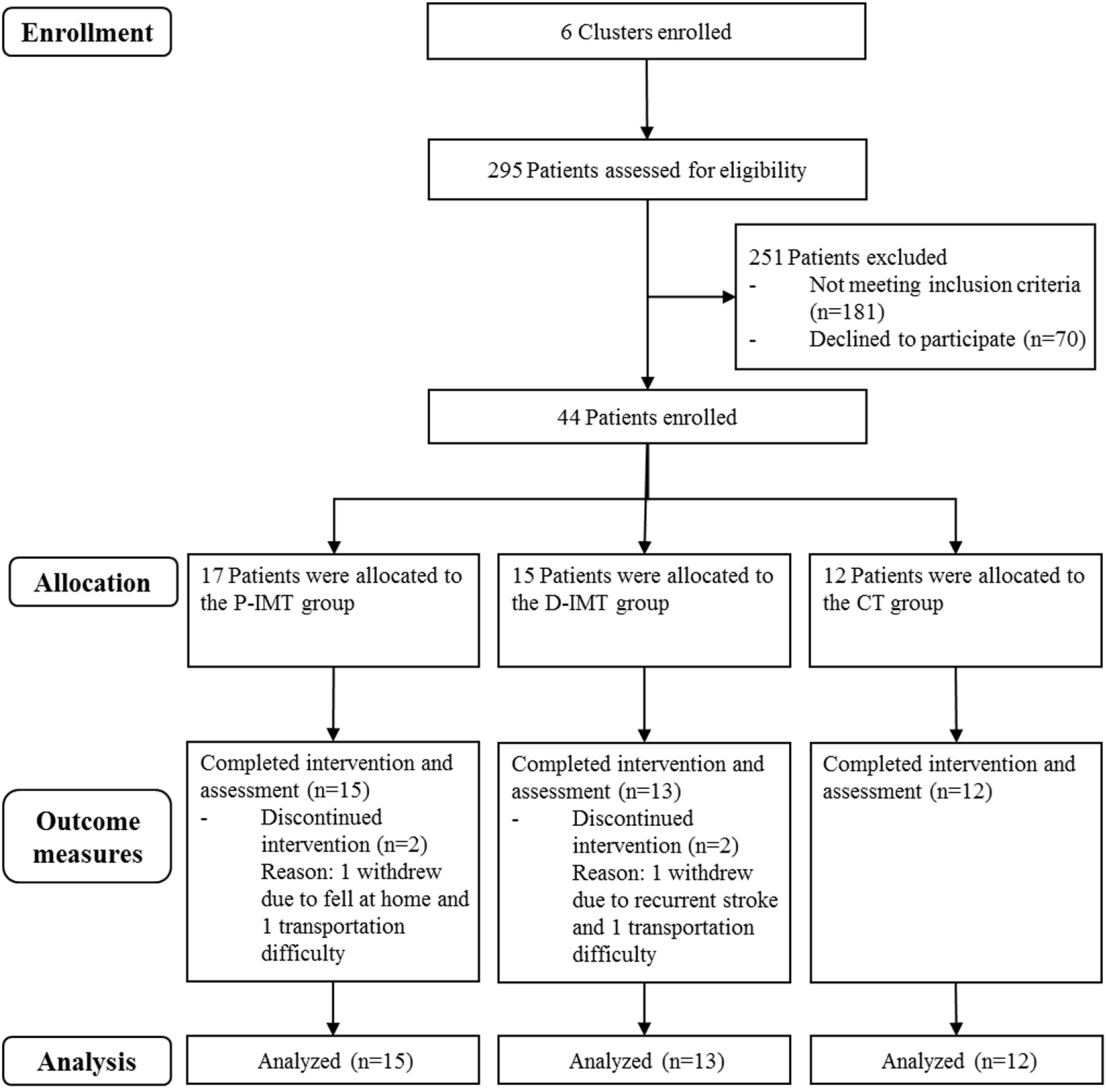
Flowchart of the participants.

This study was a cluster-controlled trial, and Fig. 1 shows the recruitment process. Because the robotic devices are not portable and cannot be easily moved, we determined a cluster design to randomize the participants in groups based on the hospitals would be a good, feasible approach^26^. Randomization was thus conducted at the cluster level rather than at the individual patient level. A cluster was defined as a hospital. This trial had at least 2 clusters for each group to avoid the treatment effects largely confounded with the cluster effect. Six hospitals participated in this study. A research assistant who was not involved in the assessment and intervention managed the randomization procedure.

The participants were screened and recruited from the occupational therapy clinics. Patients who met the eligibility criteria were invited to participate in the study by the research staff. All participants received 20 sessions of treatment (90 to 100 minutes per session, 5 days per week, for 4 weeks). Outcome measures were administered to patients at baseline and immediately after the 4-week intervention. The raters were blinded to the group assignment and did not screen participants or provide intervention. Only on the evaluation days did the raters go to the clinics to conduct the assessments.

### Interventions

The participants received 1 of the 3 interventions: P-IMT, D-IMT, or CT.

#### P-IMT protocol

**Figure 2:**
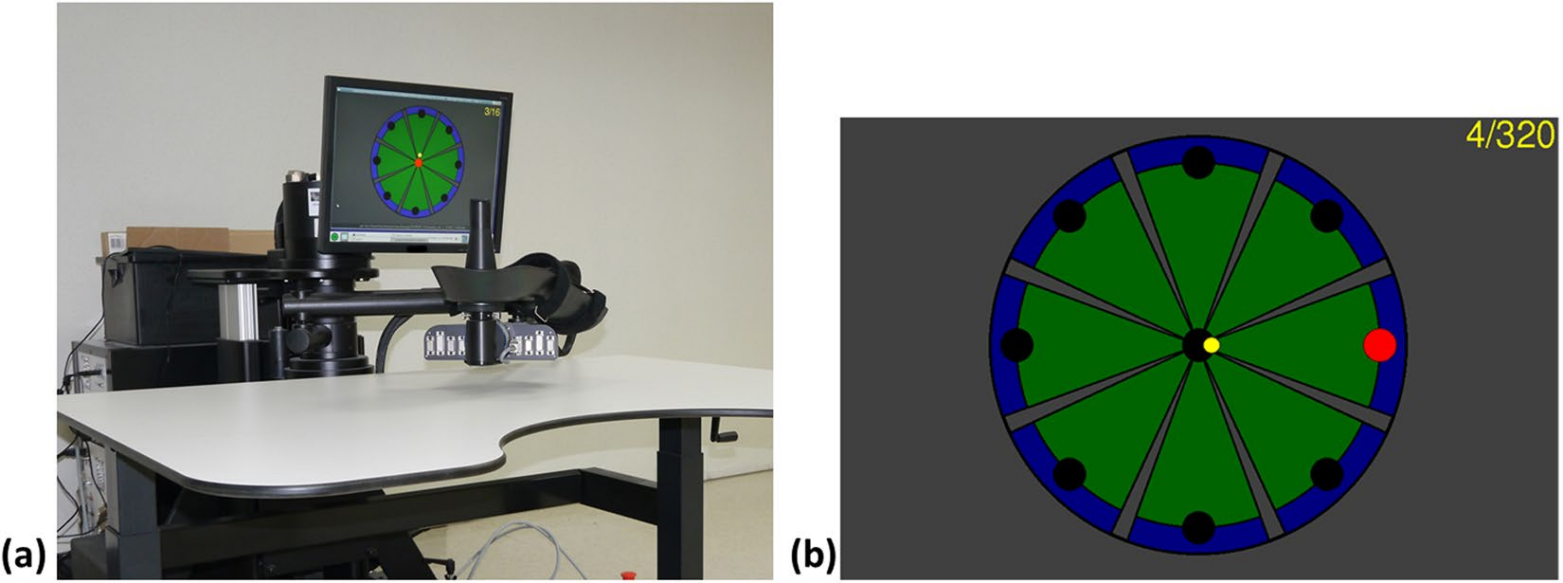
(**a**) InMotion ARM interactive therapy system. (**b**) Clock game. Eight targets were distributed along a circle on a computer screen. The clock program required the participants to move the target from the center (yellow cursor) to each of the 8 targets (red circle).

The InMotion ARM (ie, InMotion 2.0) interactive therapy system (Bionik Laboratories, Watertown, MA, USA), which consists of a shoulder-elbow unit for planar movements with 2 degrees of freedom, was used in this group (Fig. 2a). The robot had a height-adjustable workstation, and the participant was seated with the robot aligned with his or her midline. The participant held the robot handle with the affected hand, and a forearm support was provided. The height of the robot arm was adjusted so the participant’s forearm could be parallel to the floor.

The participants interacted with a clock game that displayed 8 targets distributed along a circle on a computer screen and were asked to move the target from the center to each of the 8 targets sequentially (Fig. 2b). During the clock game, the position of the end-effector held by participants was presented as a yellow cursor. A blinking red circle on the screen was the target to be reached. If the patient could move the cursor to reach the target, the next blinking red target was presented immediately. If the patient could not reach the target, a force provided by the robot assisted the patient’s movements.

The active-assisted mode was mainly applied in this study. In this mode, the robot provided assistance to help patients reach the targets when they could not perform the whole range of movement independently. The amount of assistance was adjusted and tuned based on the patient’s performance by using the robot’s built-in, performance-based control algorithm^27^. The robot measured the parameters of the algorithm while the patients performed the tasks. During each training session, the participants completed 1024 repetitions of movements within 40 to 50 minutes^6,17^. The patients received 3 series of 320 assisted clockwise repetitions (*Adaptive*) and 4 series of 16 unassisted clockwise repetitions (*Record*) of movements. One clockwise repetition indicated a point-to-point movement (eg, from the center to the target). The therapist provided instructions and general encouragement, and the patients also received specific performance feedback on the screen.

According to the patient’s levels of motor ability, other modes, such as passive, active, or resistive modes, could also be applied. If the patient could not reach more than half of the targets during first 16 unassisted movements, the following one or two series of 320 assisted movements was replaced by the passive mode. If the patient could complete the game task during first 16 unassisted movements and exhibit small movement jerks in the active-assisted mode, one or two series of 320 assisted movements was replaced by the active or resistive mode.

Approximately 40 to 45 minutes of functional task practice was provided after RT. The functional tasks used in this part of the training included tool use, dressing, reading a magazine, folding a towel, wiping a table, meal preparation, and using a cellular phone. Task difficulty was adjusted according to the patient’s level of motor ability, individual needs, and progress. Some general ways to grade the selected tasks were adopted: changing the position of task materials (eg, height, distance, or direction), changing the weight or size of objects, changing the complexity/step of tasks, and changing the levels of manual dexterity.

#### D-IMT protocol

**Figure 3:**
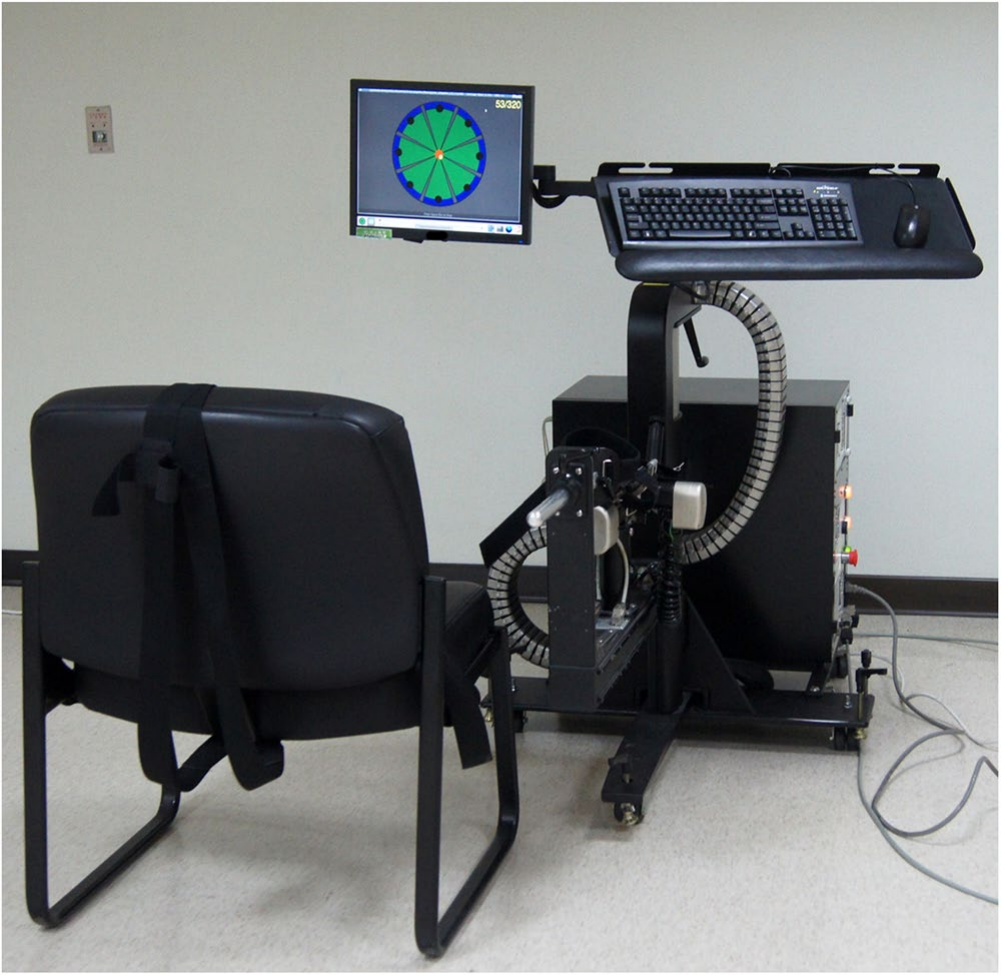
InMotion WRIST interactive therapy system.

The InMotion WRIST (ie, InMotion 3.0) interactive therapy system (Bionik Laboratories, Watertown, MA, USA) was used to execute wrist and forearm movements in this group (Fig. 3). The patients held the robot with the affected hand and placed their forearm into an arm trough support. During each session, the participants also completed 1024 repetitions of wrist and forearm movements within 40 to 50 minutes. The clock game and point-to-point game in the InMotion WRIST were mainly used. The patient performed wrist flexion/extension and wrist abduction/adduction during the clock game and performed forearm pronation/supination movements during the point-to-point game. The active-assisted mode was mainly applied in these games, but other modes (eg, passive, active, or resistive modes) could also be used based on the patient’s levels of motor ability. The principle of mode adjustment was the same as in the P-IMT group.

Other treatment principles of this group were the same as those performed in the P-IMT group, such as providing feedback, and 40 to 45 minutes of functional task practice following the RT intervention.

#### CT protocol

The participants in the CT group received 45 minutes of conventional rehabilitation and 45 minutes of functional task practice per session. Customary and traditional techniques and programs provided in the conventional rehabilitation session were passive or active range of motion exercise, gross motor training, object manipulation or fine motor training, and muscle strengthening. The treatment principles and programs of the functional task practice session were the same as those conducted in the 2 RT groups.

### Primary outcome measures

#### Fugl-Meyer Assessment (FMA) - Upper Limb Subscale

The FMA, which is administered by a skilled examiner, assesses the patient’s reflexes, movements, and coordination of UL segments^28^. It consists of 33 items, and the total score ranges from 0 to 66, with higher scores indicating better motor function. The FMA can be also separated into the subscores of a proximal unit of shoulder/elbow (0 to 42) and a distal unit of hand/wrist (0 to 24) to represent proximal and distal UL scores, respectively. The psychometric properties of the total FMA score and distal FMA subscale have been well established^29–32^.

#### Medical Research Council (MRC) scale

The MRC scale, which is a reliable measurement in stroke patients^33^, was used to examine muscle strength of the flexors and extensors of the shoulder, elbow, wrist, and fingers of the patient’s affected arm. The score ranges from 0 to 5, with higher scores indicating greater muscle strength. This study analyzed the proximal and distal MRC scores in addition to the total score and reported the average MRC scores.

### Secondary outcome measures

#### Motor Activity Log (MAL)

The MAL is a self-report scale that assesses how patients rate the amount of use (MAL-AOU) and quality of movement (MAL-QOM) of using their affected arm during 30 daily functional activities^34^. The score of each item ranges from 0 to 5, and higher scores indicate more frequently used or higher quality of movement. The MAL has been validated as a good, valid, and responsive scale^35,36^.

#### Wrist-worn accelerometers

ActiGraph GT3X^+^ accelerometers were used to provide an objective measure of the amount the affected arm was used in the patient’s real-life situation^37^. The participants wore the triaxial ActiGraph GT3X^+^ on each wrist for 3 consecutive days, before and after treatment. The ActiGraph accelerometers were only used in the P-IMT and D-IMT groups because of a limited number of devices. The ActiLife 6.10 software (ActiGraph, Pensacola, FL, USA) was used to process acceleration data. The raw data were integrated into 60-second epochs. The average counts of physical activity in the affected arm and the ratio of activity counts between the affected and nonaffected arms were reported.

### Statistical analysis

The χ^2^ test for categoric variables and 1-way analysis of variance for continuous variables were used to compare the baseline characteristics of the 3 groups. Analysis of covariance was used to evaluate the differences in the outcomes among the 3 treatment groups, with the pretreatment scores as the covariates. The Bonferroni correction was used for post hoc comparisons. The effect size of partial eta squared was also calculated to index the magnitude of difference. Partial eta squared is a commonly reported effect size measure with analysis of covariance and is calculated as the ratio of the between-group sum of squares to the sum of between-group and error sum of squares^38^. A large effect is represented by a partial eta squared of at least 0.138, a moderate effect by 0.059, and a small effect by 0.010^39^. Statistical analyses were performed with SPSS 19 software (IBM Corp, Armonk, NY, USA). A *P* value of ≤0.05 indicated statistical significance.

Previous data^6,12,40^ examining the effects of RT on the FMA were used to estimate an effect size partial eta squared of 0.29 to 0.51 for a study design of 3-group comparisons. To reach a power of 0.80 and a 2-sided type I error of 0.05, 10 to 36 patients per group had to be recruited if the randomization were at the individual patient level. Because the observations on subjects in the same cluster might be correlated, the required sample size for a cluster study design needed to be increased^26^. The increased sample size depends on the average cluster size and the degree of correlation within clusters. Because the randomization was at the cluster level in this study, the required sample size was increased from 12.5 to 45.0 patients per group (ie, inflation factor of 1.25), given an assumed intracluster correlation of 0.05^26^, 6 patients in each cluster, and 6 participating clusters^41^.

## Results

### Participants’ baseline characteristics

The study enrolled 6 hospitals in North Taiwan from September 2014 to July 2016, and 40 patients with stroke completed the study intervention and assessment (Fig. 1), with 15 patients in the P-IMT group, 13 in the D-IMT group, and 12 in the CT intervention. Patients were a mean age of 54.42 years, and the average time after stroke onset was 20.58 months. No statistically significant differences were found for the baseline characteristics of the participants among the 3 groups (Table 1).

**Table 1:** Baseline demographic and clinical characteristics of the participants.

Variable	P-IMT (n = 15)	D-IMT (n = 13)	CT (n = 12)	*P* value
Gender, No.				0.29
Male	13	8	8	
Female	2	5	4	
Age, mean (SD), y	57.27 (12.94)	50.35 (16.65)	55.27 (10.50)	0.40
Months after stroke, mean (SD)	21.67 (11.88)	14.92 (6.59)	25.33 (17.46)	0.12
Side of brain lesion, No.				0.58
Left	7	8	5	
Right	8	5	7	
Type of stroke, No.				0.72
Hemorrhage	8	5	6	
Ischemic	7	8	6	
Lesion site, No.				0.90
Cortical	6	6	6	
Subcortical	8	6	6	
Others	1	1	0	
Handedness, No.				0.43
Right	14	13	12	
Left	1	0	0	
Year of education, mean (SD)	10.67 (5.88)	12.65 (3.08)	12.08 (3.09)	0.47
MMSE, mean (SD)	27.40 (2.26)	28.54 (1.45)	27.58 (2.39)	0.32
FMA, mean (SD)	35.13 (10.39)	36.77 (7.52)	29.58 (9.13)	0.14
MRC, mean (SD)	3.12 (0.86)	2.82 (0.68)	2.33 (0.91)	0.06

CT, control treatment; D-IMT, distal-emphasized InMotion WRIST system; FMA, Fugl-Meyer Assessment; MMSE, Mini-Mental State Examination; MRC, Medical Research Council Scale; P-IMT, proximal-emphasized InMotion ARM system; SD, standard deviation.

### Effects on the primary measures

**Figure 4:**
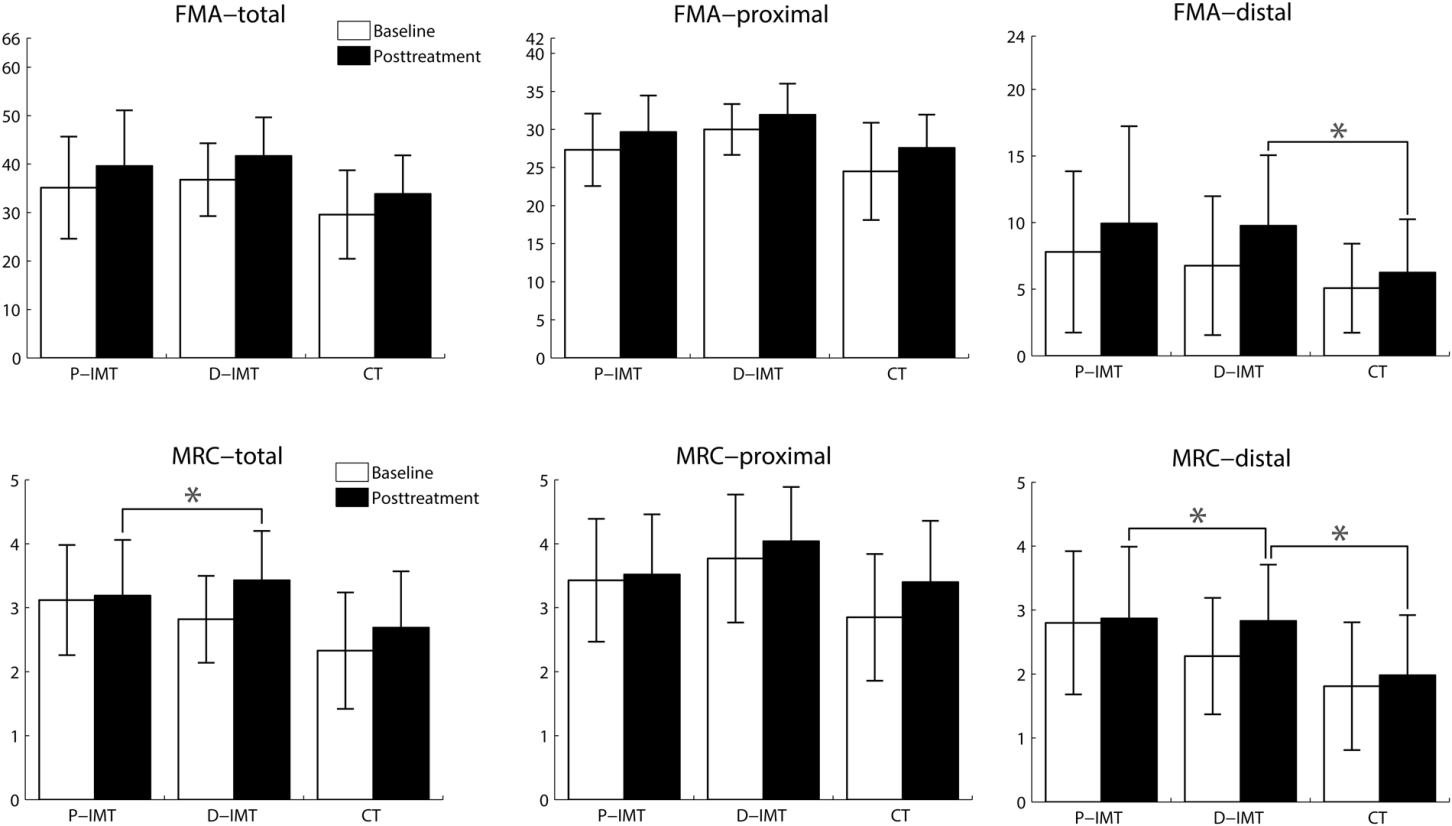
The baseline and posttreatment scores of the 3 groups on the primary outcomes. *P ≤ 0.05. On the FMA-distal, the D-IMT group had a significant improvement compared with the CT group. On the MRC-total, the D-IMT group improved significantly compared with the P-IMT group. On the MRC-distal, the D-IMT group showed significantly greater improvements than the P-IMT and CT groups.

For the primary outcomes, there were statistically significant differences and large effect sizes on the distal FMA, total MRC, and distal MRC scores among the 3 groups (*P* = 0.02 to 0.04, Table 2). Post hoc analyses showed that the D-IMT group had a significantly better outcome than the P-IMT group on the total MRC (*P* = 0.04) and distal MRC (*P* = 0.04) (Fig. 4). The D-IMT group also showed significantly greater improvements than the CT group on the distal FMA (*P* = 0.03) and distal MRC (*P* = 0.04) (Fig. 4). The differences on the FMA and MRC between the P-IMT and CT groups were not statistically significant (*P*s > 0.77). The D-IMT group showed the most improvements in the distal part of UL motor function and muscle strength among the 3 intervention groups. In addition, there were no significant differences among the 3 groups on the total FMA (*P* = 0.77), proximal FMA (*P* = 0.97), and proximal MRC (*P* = 0.12) scores.

**Table 2:** Descriptive statistics and group comparisons on the primary outcomes.

Outcome	P-IMT Mean (SD)	D-IMT Mean (SD)	CT Mean (SD)	ANCOVA		
				*F*	*P*	Partial eta squared
FMA-total (0–66)				0.27	0.77	0.02
Baseline	35.13 (10.52)	36.77 (7.52)	29.58 (9.13)			
Posttreatment	39.60 (11.49)	41.69 (7.96)	33.83 (7.98)			
Mean difference (95% CI)	4.47 (3.21–5.72)	4.92 (3.28–6.57)	4.25 (2.81–5.69)			
FMA-proximal (0–42)				0.04	0.97	< 0.01
Baseline	27.33 (4.75)	30.00 (3.34)	24.50 (6.38)			
Posttreatment	29.67 (4.79)	31.92 (4.09)	27.58 (4.36)			
Mean difference (95% CI)	2.34 (1.25–3.41)	1.92 (0.95–2.89)	3.08 (1.26–4.91)			
FMA-distal (0–24)				3.95	0.03*	0.18
Baseline	7.80 (6.05)	6.77 (5.21)	5.08 (3.34)			
Posttreatment	9.93 (7.30)	9.77 (5.29)	6.25 (4.00)			
Mean difference (95% CI)	2.13 (1.13–3.13)	3.00 (2.01–3.99)	1.17 (0.51–1.82)			
MRC-total (0–5)				3.50	0.04*	0.16
Baseline	3.12 (0.86)	2.82 (0.68)	2.33 (0.91)			
Posttreatment	3.19 (0.87)	3.43 (0.77)	2.69 (0.88)			
Mean difference (95% CI)	0.07 (–0.05 to 0.20)	0.61 (0.13–1.10)	0.36 (0.15–0.56)			
MRC-proximal (0–5)				2.21	0.12	0.11
Baseline	3.43 (0.96)	3.77 (1.00)	2.85 (0.99)			
Posttreatment	3.52 (0.94)	4.04 (0.85)	3.40 (0.96)			
Mean difference (95% CI)	0.09 (–0.12 to 0.28)	0.27 (–0.05 to 0.59)	0.55 (0.20–0.88)			
MRC-distal (0–5)				4.71	0.02*	0.21
Baseline	2.80 (1.12)	2.28 (0.91)	1.81 (1.00)			
Posttreatment	2.87 (1.12)	2.83 (0.88)	1.98 (0.94)			
Mean difference (95% CI)	0.07 (–0.08 to 0.21)	0.55 (0.24–0.87)	0.17 (–0.14 to 0.47)			

ANCOVA, analysis of covariance; CI, confidence interval; CT, control treatment; D-IMT, distal-emphasized InMotion WRIST system; FMA, Fugl-Meyer Assessment; MRC, Medical Research Council Scale; P-IMT, proximal-emphasized InMotion ARM system; SD, standard deviation.

*P ≤ 0.05.

### Effects on the secondary measures

**Figure 5:**
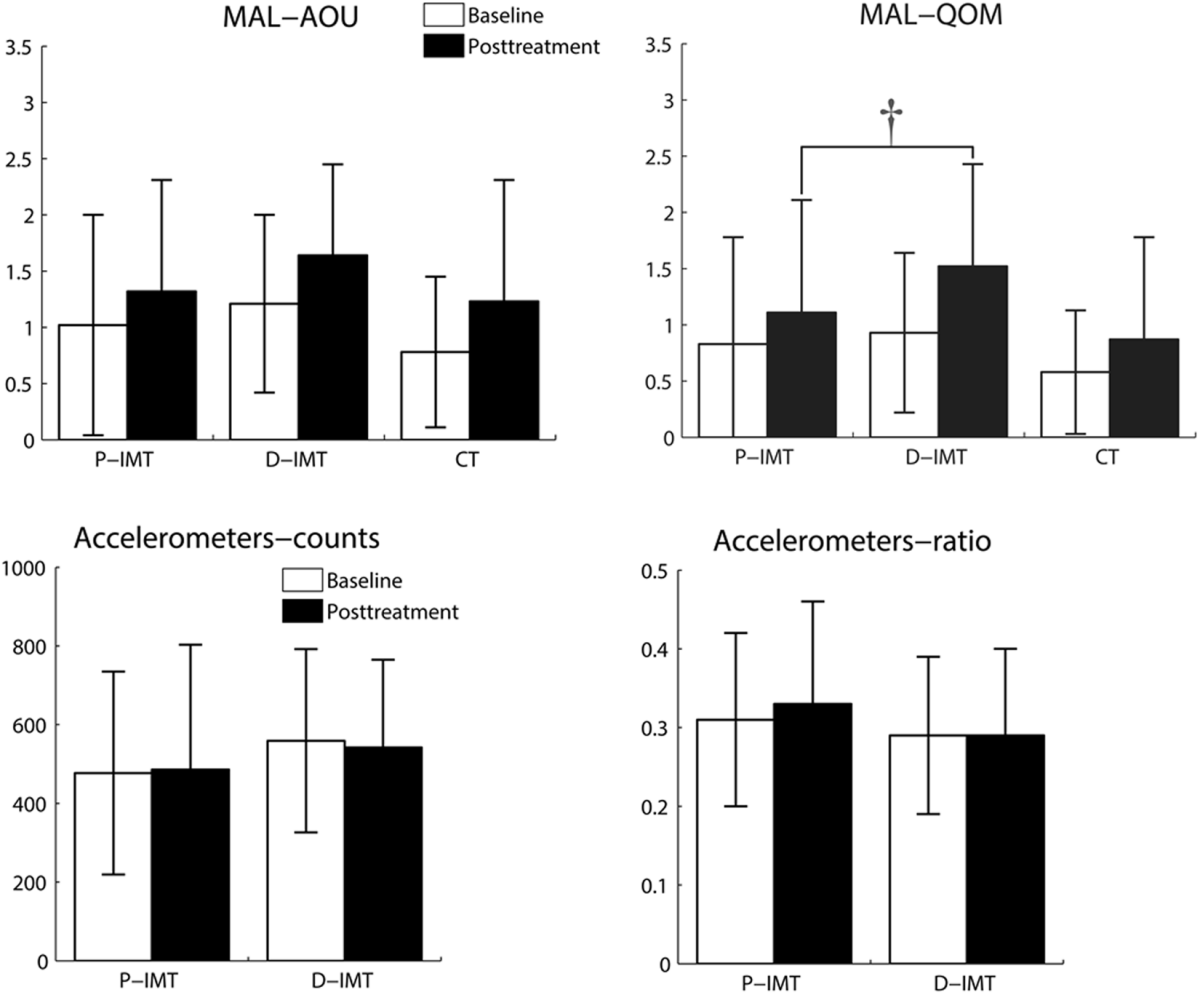
The baseline and posttreatment scores of the 3 groups on the secondary outcomes. ^†^*P* = 0.06. On the MAL-QOM, the D-IMT group had approached significantly higher improvement than the P-IMT group.

As reported in Table 3 for the secondary outcomes, a statistically significant difference among the 3 groups was found on the MAL-QOM (*P* = 0.05). Post hoc analyses revealed that the D-IMT group had approached significantly higher improvement in the MAL-QOM than the P-IMT group (*P* = 0.06) (Fig. 5). However, the differences of MAL-AOU among the 3 groups were comparable and not statistically significant (*P* = 0.57) (Table 3). In addition, the ratios of affected to nonaffected arm use in the participants were nearly 30%. There were no significant differences on the activity counts of the affected arm (*P* = 0.32) and the ratio between both arms (*P* = 0.09) detected by the accelerometers between the P-IMT and D-IMT groups (Fig. 5).

**Table 3:** Descriptive statistics and group comparisons on the secondary outcomes.

Outcome	P-IMT Mean (SD)	D-IMT Mean (SD)	CT Mean (SD)	ANCOVA		
				*F*	*P*	Partial eta squared
MAL-AOU (0–5)				0.58	0.57	0.03
Baseline	1.02 (0.98)	1.21 (0.79)	0.78 (0.67)			
Posttreatment	1.32 (0.99)	1.64 (0.81)	1.23 (1.08)			
Mean difference (95% CI)	0.31 (0.15–0.46)	0.43 (0.29–0.56)	0.45 (0.08–0.81)			
MAL-QOM (0–5)				3.29	0.05*	0.15
Baseline	0.83 (0.95)	0.93 (0.71)	0.58 (0.55)			
Posttreatment	1.11 (1.00)	1.52 (0.91)	0.87 (0.91)			
Mean difference (95% CI)	0.28 (0.18–0.38)	0.59 (0.38–0.80)	0.29 (0.00–0.58)			
Accelerometers^†^						
Counts				1.03	0.32	0.04
Baseline	477.17 (257.84)	559.11 (232.88)	—			
Posttreatment	485.77 (317.56)	542.02 (223.02)	—			
Mean difference (95% CI)	8.60 (−43.20 to 60.39)	−17.09 (−59.71 to 25.53)	—			
Ratio				3.13	0.09	0.12
Baseline	0.31 (0.11)	0.29 (0.10)	—			
Posttreatment	0.33 (0.13)	0.29 (0.11)	—			
Mean difference (95% CI)	0.02 (0.00–0.04)	0.00 (−0.01 to 0.01)	—			

ANCOVA, analysis of covariance; AOU, amount of use; CI, confidence interval; CT, control treatment; D-IMT, distal-emphasized InMotion WRIST system; MAL, Motor Activity Log; P-IMT, proximal-emphasized InMotion ARM system; SD, standard deviation; QOM, quality of movement.

*P ≤ 0.05.

^†^Accelerometers were only administered to the P-IMT (n = 15) and D-IMT (n = 12) groups due to limited devices.

### Monitoring of adverse effects

There were no treatment-related serious adverse events. Events unrelated to study therapy were that 1 patient fell at home and 1 patient had a recurrent stroke (Fig. 1). In addition, the mean pain and fatigue ratings of the 3 intervention groups at the first and last therapy sessions were 1.0 to 2.9 on a scale of 0 to 10. The patients reported mild pain mainly due to muscle soreness or stiffness. The pain scores of most patients were maintained or decreased from the first to the last day, indicating that the study interventions did not increase pain of patients.

## Discussion

The participants receiving D-IMT had greater improvements in muscle strength and quality of movement while using the affected arm in daily activities than those receiving the P-IMT. The D-IMT group showed better outcomes in distal UL motor function and distal muscle strength compared with the CT intervention. In addition, the amount of improvement on the total FMA score (ie, 5 points) in both the D-IMT and P-IMT groups was similar to the gains reported in several previous RT studies using the same robotic devices^6,42,43^. The improved scores of the D-IMT and P-IMT groups on the total FMA also reached the threshold of minimal clinically important difference^44^. In addition, the P-IMT group did not show advantages over the D-IMP and CT interventions on the assessments of proximal UL motor function and muscle strength, indicating that the 3 groups demonstrated similar benefits on the proximal outcomes.

The D-IMT group gained the most improvements in the distal part of UL motor function and muscle strength and in quality of movement while using the affected UL in daily activities among the 3 groups. The change scores for 9 (69.2%), 6 (46.2%), 8 (61.5%), and 7 (53.8%) participants in the D-IMT group exceeded the minimal clinically important difference values (ie, 10% of total score)^45^ of the FMA-distal, MRC-total, MRC-distal, and MAL-QOM, respectively. The results support that these improvements after distal-emphasized RT were not only statistically significant but also clinically important. The movements of distal UL segments are crucial for executing functional tasks. The benefits of distal-emphasized RT using the InMotion WRIST seems directly related to the distal UL segments treated, implying the effects of training specificity. The more distal UL training led to greater gains in distal motor function, muscle strength, and quality of movement during functional activities.

No significant advantages of the proximal-emphasized RT using the InMotion ARM were highlighted on proximal motor function and muscle strength outcomes compared with the other 2 groups. One possible reason is that regardless of the intervention groups, the participants still used their proximal UL segments or exerted strength during treatment, albeit the upper arm was supported or restrained in the D-IMP group. The proximal and distal UL segments were also both used in some treatment programs in the CT group, including reaching, grasping, and object manipulation. The standard clinical scales used in this study could not capture the changes of motor control or movement strategies after proximal-emphasized RT. Further studies that include kinematic measures along with clinical scales to help clarify the observed changes after robotic rehabilitation are suggested. Furthermore, 2 previous studies reported that the improvement scores of the FMA were 7.7 and 11 points after RT using the same InMotion ARM plus functional task training^46,47^. The improvements on the FMA were higher than in this study (5 points). However, the disparity may have resulted from the larger treatment dosage provided in these 2 studies (5 hours a day, 5 days per week for 12 weeks) and patients with different severities of stroke recruited in the studies (moderate to severe versus mild to moderate impairment).

A previous prelimary study investigated the efficacy of the RT training sequence to different UL segments^17^. The authors examined the effects of proximal UL training, followed by distal UL and vice versa, by using the same InMotion ARM and WRIST robot systems. Their initial results showed that there were local effects on the treated UL segments but that the generalization of gains to the untreated UL segments was limited. The gains of training on the distal UL segment appeared to have a greater transfer to the proximal UL segment than vice versa^17^. Another study reported the effects of additionally adding distal RT to proximal RT, suggesting that there were no advantages on the proximal UL^22^. Moreover, 2 recent review articles^15,16^ found that proximal RT studies have a positive trend to improve UL motor function, whereas the effects of distal RT remain unclear due to low numbers of distal RT studies. Except for comparing the relative effects of proximal and distal UL training by robotic rehabilitation technology, the degrees of generalization effects to the untreated limb segments or which training order is better to achieve more recovery warrant further investigation.

Moreover, the accelerometry ratios of the affected arm to the nonaffected arm were approximately 0.3 in both RT groups. This finding was similar to previous studies that provided different UL therapies in stroke patients and also reported accelerometry ratios of 0.31 to 0.58^48–50^. These results indicate that the amount of activity in the affected arm is one-third to one-half that of the nonaffected arm. However, these studies have shown significant and nonsignificant changes in the ratios or the activity counts of the affected arm after treatment^48–50^. The accelerometry data are affected by the type of monitor that is used, the patients’ lifestyles or regular activities, the duration of data collection, and the duration of data processing. The wearable monitors provide an objective and quantitative measure of patients’ activity in real-life environments, but their clinical usefulness as an outcome measure needs to be further evaluated.

This study has some limitations. First, long-term follow-up evaluations were not performed, and thus, carry-over beneficial effects could not be determined. Second, this study was a cluster-controlled trial with a modest sample size. Further larger-scale studies are suggested to include more clusters (eg, 4 clusters per intervention arm)^26,51^ and participants (eg, 30 patients per arm) to validate the study findings. Third, although the between-group differences in baseline variables were not significant, the D-IMT group had shorter onset time and higher baseline FMA and MMSE scores, which may have biased the outcomes. The differential effects of proximal versus distal UL robotic rehabilitation in the patients with more severe motor deficits (eg, FMA < 18) warrant further investigations. In addition to the wrist robotic device applied in this study, further research that includes hand/digit robotic devices (eg, InMotion Hand Robot) is suggested to provide a more comprehensive efficacy of distal UL robotic rehabilitation.

In conclusion, we found that the distal-emphasized RT had superior outcomes compared with the proximal-emphasized RT on muscle strength and quality of movement during functional activities after the 4-week intervention. The distal-emphasized RT showed better improvements than CT on distal upper-limb motor function and distal muscle strength. In addition, the improvements of the D-IMT and P-IMT groups on overall upper-limb motor function both reached the minimal clinically important difference. Further larger-scale research is suggested to investigate the long-term differential effects between proximal- and distal-emphasized robotic rehabilitation after stroke.

### Data availability statement

The data sets generated during and/or analyzed during the current study are available from the corresponding author on reasonable request.

## Electronic supplementary material

Supplementary Information
